# The Role of Normothermic Perfusion in Liver Transplantation (TRaNsIT Study): A Systematic Review of Preliminary Studies

**DOI:** 10.1155/2018/6360423

**Published:** 2018-05-17

**Authors:** Kumar Jayant, Isabella Reccia, Francesco Virdis, A. M. James Shapiro

**Affiliations:** ^1^Department of Surgery and Cancer, Imperial College London, London, UK; ^2^Department of Surgery, Kings College, London, UK; ^3^Department of Surgery, University of Alberta, Edmonton, Canada

## Abstract

**Introduction:**

The success of liver transplantation has been limited by the unavailability of suitable donor livers. The current organ preservation technique, i.e., static cold storage (SCS), is not suitable for marginal organs. Alternatively, normothermic machine perfusion (NMP) promises to recreate the physiological environment and hence holds promise for the better organ preservation. The objective of this systematic review is to provide an overview of the safety, benefits, and insight into the other potential useful parameters of NMP in the liver preservation.

**Material and Methods:**

We searched the current literature following registration in the International Prospective Register of Systematic Reviews (PROSPERO) with registration number CRD42018086034 for prospective trials comparing the role of NMP device to SCS in liver transplant by searching the PubMed, EMBASE, Cochrane, BIOSIS, Crossref, and Scopus databases and clinical trial registry.

**Results:**

The literature search identified five prospective clinical trials (four being early phase single institutional and single randomized multi-institutional) comparing 187 donor livers on NMP device to 273 donor livers on SCS. The primary outcome of interest was to assess the safety and graft survival at day 30 after transplant following NMP of the donor liver. Secondary outcomes included were early allograft dysfunction (EAD) in the first seven days; serum measures of liver functions as bilirubin, aspartate aminotransferase (AST), alanine amino transferase (ALT), alkaline phosphatase (ALP), and international normalized ratio (INR) on days 1–7; major complications as defined by a Clavien-Dindo score ≥ 3; and patient and graft survival and biliary complications at six months. The peaked median AST level between days 1 and 7 in the five trials was 417–1252 U/L (range 84–15009 U/L) while on NMP and 839–1474 U/L (range 153–8786 U/L) in SCS group. The median bilirubin level on day 7 ranged within 25–79 *µ*mol/L (range 8–344 *µ*mol/l) and 30–47.53 *µ*mol/l (range 9–340 *µ*mol/l) in NMP and SCS groups, respectively. A single case of PNF was reported in NMP group in the randomized trial while none of the other preliminary studies reported any in either group. There was intertrial variability in EAD which ranged within 15–56% in NMP group while being within 23–37% in SCS group. Biliary complications observed in NMP group ranged from 0 to 20%. Single device malfunction was reported in randomized controlled trial leading to renouncement of transplant while none of the other trials reported any machine failure, although two user related device errors inadvertent were reported.

**Conclusion:**

This review outlines that NMP not only demonstrated safety and efficacy but also provided the favourable environment of organ preservation, repair, and viability assessment to donor liver prior to the transplantation with low rate of posttransplantation complication as PNF, EAD, and biliary complication; however further studies are needed to broaden our horizon.

## 1. Introduction

**Figure 1:**
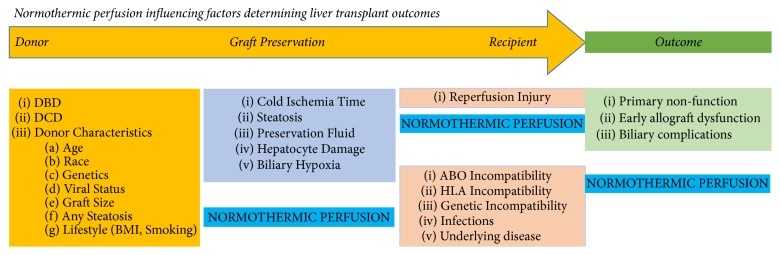
Factors modified by normothermic perfusion during liver transplantation.

Liver transplantation is established as the treatment of choice for patients with the end-stage liver disease. While the success of liver transplantation is unquestioned, the scarcity of donor organs limits the delivery of this therapy in a sufficiently timely manner to prevent deaths on the waiting list. Despite the rise in organ donation, the potential requirement of liver transplantation still far exceeds demand, and patients may have compromised outcomes as they end up being transplanted with high model of end-stage liver disease (MELD) scores and in a severely deconditioned state [1, 2]. The United States Organ Procurement and Transplantation Network 2016 national data found that 1,104 patients died while waiting for a liver transplant and a further 1,317 were removed from the list as they became too sick to transplant [3]. The global escalating shortage of organ donors has driven centres to use extended criteria donors (ECD), including elderly people, steatotic liver, and donation after cardiac death (DCD), as well as recently using donors that are actively infected with hepatitis C virus. However, inadequate liver preservation and extensive ischemic injury in ECD grafts have been recognized as key factors associated with primary non function (PNF), early allograft dysfunction (EAD), and biliary complications [4, 5]. If more marginal and ECD livers could be preserved with a system that could protect and reverse hepatocyte and biliary injury, without excess risk in transplanting, then potentially the supply and demand for liver transplantation could become more manageable (Figure 1) [1, 6].

The acute termination of oxygenated blood supply during liver procurement initiates a cascade of injury and inflammation, triggered initially by hypoxic anaerobic metabolism, nutrient, cofactor, and adenosine triphosphate (ATP) depletion, with lactic acid accumulation. These injurious processes are further exaggerated during static cold storage (SCS). Disruption of sodium-potassium membrane pumps leads to disruption of electrolyte cell membrane gradients, resulting in cellular edema, influx of free calcium, and subsequent activation of proteolytic enzymes terminating with cell death. Accumulation of xanthine oxide following ATP breakdown generates free radical upon restoration of circulation. These free radicals lead to lipid peroxidation and cellular destruction known as ischemia-reperfusion injury [7–9].

The traditional method of organ preservation involves flushing of cold preservation solution following complete dissection and interruption of blood supply to the donor organ. Cold preservation works on the principle of diminishing cellular metabolism with a decrease in temperature (the Q10 effect), which limits need for ATP [10]. However, anaerobic metabolism continues albeit slowly down to temperatures of +1°C, which can lead to continual depletion of ATP reserve and accumulation of metabolic waste. These insults are exaggerated in marginal livers, increasing risk of initial poor function (IPF), primary nonfunction (PNF), and biliary complications including ischemic cholangiopathy (IC) in comparison to standard criteria donors [11–13]. The increasing level of societal obesity and associated steatosis pose their own additional challenges, as such livers already have increased risk of IPF [14, 15]. Such marginal livers are especially vulnerable to ischemia-reperfusion injury [16, 17]. Hence, graft reperfusion may induce acute metabolic stress and give rise to hemodynamic instability known as “postreperfusion syndrome (PRS)” [18, 19]. PRS is defined as a decrease in the mean arterial pressure (MAP) of more than 30% from baseline measure recorded during the anhepatic phase, lasting for more than one minute, within the first five minutes of the reestablishment of graft perfusion [20]. The incidence of PRS varies between 25 and 50% and is associated with increased risk of acute kidney injury and increased risk of death [21–23].

The possibility of avoiding cold ischemic injury altogether in marginal grafts has recently become possible with the introduction of the new technology of ex vivo normothermic liver perfusion (NMP). The concept of normothermic perfusion is simple, in that maintaining an entirely physiological milieu for the liver during transport should diminish risk of ischemic injury within the liver, and ischemia-reperfusion has already occurred on the device, thereby reducing risk of PRS in the recipient [24]. It remains to be seen, however, if elimination of the cold ischemic phase in DCD and other marginal grafts can protect against IC and other biliary complications through application of NMP [25–27]. If reliable predictive markers of posttransplant function can be established during the NMP phase, then livers at highest risk of PNF and IPF could be eliminated before exposing a recipient to a higher risk of demise. By selecting an increasing number of livers based on ex situ function and eliminating those at demonstrably higher risk, the ceiling for extended criteria liver donors could be raised considerably. The added advantage of having a donor liver function physiologically ex situ is that protective supplements may be added to the circuit with potential to stabilize, reverse, and even repair preexisting injury. Furthermore, immunological manipulation of liver grafts could mitigate need for potent antirejection therapies in the recipient if HLA Class II expression and other donor antigens could be modified. Livers could be loaded with protective cells such as regulatory T cells (Tregs) or mesenchymal stem cells (MSCs) that also have immunoregulatory and anti-inflammatory properties (Figure 1) [28–30]. NMP has already been shown to be highly effective in lung transplantation and is currently in early developmental testing in kidney and whole pancreas transplantation [31, 32].

Based upon extensive promising results in preclinical large animal studies in NMP porcine or rodent models [33–36], at least three commercial normothermic perfusion devices have emerged in early clinical trials till date. Each NMP technology works on similar principles but differs in terms of portability, degree of automation, substrate type and delivery, pressure and pulsatility of the recirculating perfusate, and hepatic arterial and portal vein flow targets [37, 38]. The first technology to reach the clinic was the Metra device developed by Peter Friend and colleagues in a partnership between the University of Oxford and a spin-off company (OrganOx Ltd.). The Metra is a fully automated, portable device, perfusing livers at 37°C with whole blood supplemented with plasma expander (Gelofusine), bile salts, parenteral nutrition solution, heparin, insulin, and prostacyclin through a closed perfusion, continuous, nonpulsatile portal vein, and hepatic arterial flow technique [39]. The Organ Care System (OCS) liver was developed by TransMedics (Andover, MA) is also a fully automated portable device, and follows similar principles of NMP [40]. Organ Assist (Groningen, Netherlands) developed a semiautomated device but with only limited portability which allows liver perfusion at temperatures ranging from 8°C to 37°C. The arterial and portal pressures can be modulated to adjust vascular flow [41]. The importance of device portability remains an open question presently. Provision of on-board oxygen generation and a need to transport heavy, complex equipment great distances by road or plane pose their own unique challenges and markedly escalate the cost of the technology. Some institutes are now exploring the more limited intervention of perfusing the liver once it has arrived at the recipient centre. While this may not completely protect against hepatocyte injury and IC, it still offers a promising role in confirming that a liver will function before it is transplanted and offers the added advantage of being able to be more flexible to schedule liver transplantation surgeries during daylight hours.

In the present systematic review, we provide a detailed analysis of all available human liver NMP studies that assess safety, feasibility, and reliability of this new technology and where possible available evidence reflecting the clinical effectiveness of NMP as an alternative to SCS in patients undergoing liver transplantation is summated. Finally, we explored potential directions for future research and translation of NMP technique into clinical practice.

## 2. Material and Methods

### 2.1. Search Strategy

A comprehensive systematic literature review was performed according to the Preferred Reporting Items for Systematic reviews and Meta-Analyses for Protocols 2015 (PRISMA-P 2015) [46], following registration in the International Prospective Register of Systematic Reviews (PROSPERO) [47], at https://www.crd.york.ac.uk/prospero with registration number CRD42018086034. An extensive search of all the published literature describing the role with NMP based device in liver transplantation as an alternative to SCS was made on National Library of Medicine Database, EMBASE, Cochrane, BIOSIS, Crossref, and Scopus databases and clinical trial registry on 10 October 2017. The search covered the period from May 2016 (the year of the first reported clinical trial of NMP based device) to 18 April 2018 and search was last carried out on 18 April 2018 [39]. Our search strategy comprised compiling keywords as “Normothermic Perfusion”, “OrganOx”, “Organ Assist”, “Organ Care System”, “Graft Rejection”, “Graft Survival”, and “Liver Transplantation” from all the salient articles and broad literature searches on the given databases.

### 2.2. Inclusion and Exclusion Criteria

Only studies which systematically and quantitatively assessed the graft safety, functioning, and graft survival on NMP based devices including the OrganOx (Metra), the Organ Assist (Liver Assist), and TransMedics (OCS) in different clinical studies were analyzed. All other publications as editorials, reviews, and letters were excluded. The primary outcome of interest was to assess the safety and graft survival at day 30 after transplant following NMP of the donor liver. Secondary outcomes included were early allograft dysfunction (EAD) on the first seven days; serum measures of liver functions as bilirubin, aspartate aminotransferase (AST), alanine amino transferase (ALT), alkaline phosphatase (ALP), and international normalized ratio (INR) on days 1–7; major complications as defined by a Clavien-Dindo score ≥ 3; and patient and graft survival and biliary complications at six months (Table 1).

### 2.3. Data Extraction

**Figure 2:**
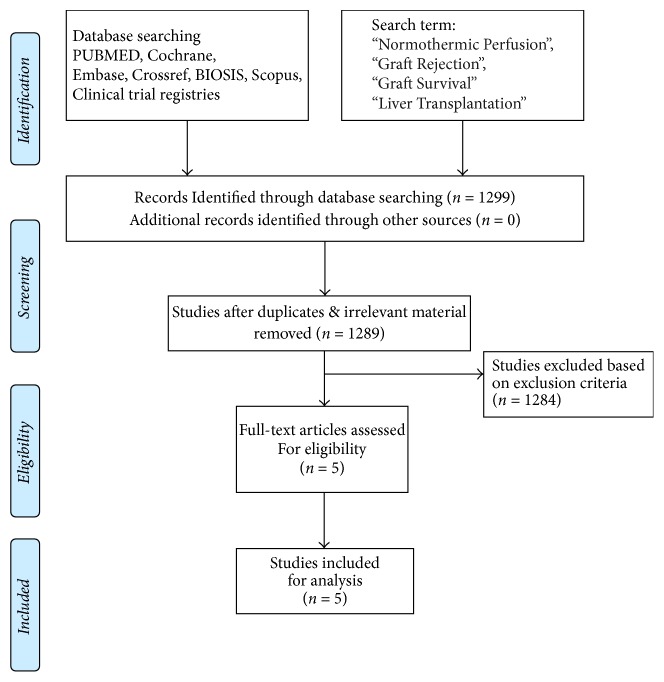
Search strategy and study selection used in this systematic review as per PRISMA protocol.

Two separate physician reviewers, KJ and IR, employed a two-stage method to conduct study screening independently. At the first stage, titles and abstracts were scrutinized for excluding obviously ineligible studies. At the second stage, the full text was read carefully for further excluding ineligible studies. Disagreements were resolved via consensus and discussion with chief author AMJS. We analyzed literature with empirical studies using a standardized quality assessment tool and prespecified inclusion and exclusion criteria. The present systematic review was performed using the Preferred Reporting Items for Systematic Reviews and Meta-Analyses (PRISMA) guidelines and registered in the PROSPERO, an international database of prospectively registered systematic reviews (Figure 2).

### 2.4. Quality Testing

The QUADAS-II (quality assessment of diagnostic accuracy studies-II) based analysis was done to assess the internal validity of prespecified inclusion and exclusion criteria of the various studies. QUADAS-II is an evidence-based bias assessment tool to evaluate the quality of diagnostic accuracy studies in a systematic review. Each study was reviewed comprehensively and data extracted to assess the earlier outlined parameters (Table 2).

### 2.5. Publication Bias

Publication bias is formally assessed through funnel plots, but that requires at least ten trials; unfortunately present systematic review involved only five trials, so we could not have assessed publication bias.

## 3. Results

Our literature search yielded a total of 1299 manuscripts using keywords listed above. After screening titles and abstracts, 5 studies (4 full articles and single poster with limited data) were included in present review analysis, data extraction of four of which involved Metra device and single with OCS (TransMedics) [39, 42–45] (Table 2). Two studies, published insofar with Organ Assist (Liver Assist) device, were not included because one of them was done at temperature of 20°C while the other did not have any control group [48, 49]. However, we did include the safety issues outlined in the article [49]. Four included studies were single institutional, nonrandomized prospective phase 1 clinical trials [39, 42–44], while study by Nasralla et al. was multi-institutional randomized study [45, 50].

The detailed data related to study characteristics in terms of perioperative and normothermic perfusion, clinical outcomes, safety, adverse events, and survival were summarized in Tables 2–6.

### 3.1. Donor and Recipient Perioperative Characteristics

A total of 460 patients were included in the five trials; we have outlined the demographic and clinical data of patients undergoing liver transplantation following organ preservation by NMP or SCS. NMP based organ preservation was done in 187 cases, while the conventional method of cold storage was used in 273 cases. Nasralla et al. reported that 48 donor livers were discarded (16 (11.7%) NMP, 32 (24.1%) SCS; *P* = 0.004) owing to presence of significant steatosis, increasing lactate level, cirrhosis in donor liver, WIT > 30 mins, incidental malignancy (colon and lung cancer), and device related errors [45]. The median patient age was outlined in four trials within 48.0–58.0 years (range 14–85) in NMP group and 46.0–58.5 years (range 20–86) in SCS group. The median MELD score reported in four trials ranged within 12–21 (range 6–40) and 14–23 (range 6–37), respectively, in NMP and SCS group. The range of DCD donor sources in NMP and SCS groups ranged between 20–40% and 10–27%, respectively (Table 2).

The reported median NMP time in trials varied considerably, from 558 to 786 minutes (range 210–1631 minutes) while median CIT on cold storage was 235–634 minutes (range 64–967 minutes). There was no significant difference in organ preservation time in the study by Ravikumar et al., Selzner et al., and Liu et al. [39, 42, 44]. However, Bral et al. noted a significantly more prolonged total liver preservation time (786 minutes NMP versus 235 minutes SCS, *P* < 0.001) between study groups [43]. They further commented in supplementary data that they extended NMP duration to enhance operating room logistics, apparently without compromising clinical outcomes for the liver. Similarly, Nasralla et al. did report significantly prolonged total liver preservation time (714 minutes NMP versus 465, *P* < 0.000) [45] (Table 2).

The attributes to assess donor liver functioning while on NMP such as hepatic transaminases, INR, pH, lactate level, bile production, hepatic artery, and portal vein flow were found normal in all the reviewed trials. The perfusate used in all studies was blood based using ABO-blood group O packed red blood cells; however, in the studies by Ravikumar et al., Bral et al., and Nasralla et al., the circuit and liver were additionally primed with gelatin-based plasma expander (Gelofusine™, B. Braun, Melsungen, Germany), whereas in the study by Selzner et al. Steen solution was used instead (Table 3).

### 3.2. Clinical and Laboratory Outcomes of Normothermic Ex Vivo Perfusion (NMP) and Static Cold Storage Liver (SCS)

The peak median AST level between days 1 and 7 in the five trials was 417–1252 U/L (range 84–15009 U/L) while on NMP and 839–1474 U/L (range 153–8786 U/L) in SCS group.

Three trials reported data with median INR value, on day 7, in NMP group being 1.05–1.1 (range 0.88–1.6) and 1.03–1.1 (range 0.90–2.2) in SCS group. The median bilirubin level on day 7 was 25–79 *μ*mol/L (range 8–344 *μ*mol/L) and 30–48 *μ*mol/L (range 9–340 *μ*mol/L) in NMP and SCS groups, respectively. Only three trials outlined day 7 median ALP level, 139–245 U/L (range 40–626 U/L) in NMP group while being 147–243 U/L (range 58–743 U/L) in SCS group (Table 4).

### 3.3. Post-Liver Transplant Outcomes

The PNF was observed in a single recipient in the randomized study done by Nasralla et al. in NMP group while none of the other trials reported such event in either groups. There was intertrial variability in EAD which ranged from 10 to 56% in NMP group while being within 23–30% in SCS group. Nasralla et al. reported 93% less likelihood of developing EAD in DCD liver while on NMP rather than SCS [45]. In the randomized trial by Nasralla et al. the occurrence of PRS was reported less frequently in NMP group (15 cases) than the SCS (32 cases) which gives credence to the earlier trial of Bral et al. [43, 45].

The median intensive care unit (ICU) stay was 3–16 days (range 1–65 days) in NMP group while in SCS group the median was 3-4 days (range 0–41 days). The median hospital stay in NMP and SCS was 12–45 days (range 6–114 days) and 13–25 days (range 7–89 days), respectively. Between 10–22% and 22–37% recipients developed Clavien-Dindo score ≥ 3 in NMP and SCS groups, respectively. Biliary complications 6 months after transplant were observed in NMP group ranging from 0 to 20%. The trial by Bral et al. evidenced lower 6 months' biliary stricture in NMP group (0%) compared to 14.8% in SCS; however a recent randomized study published by Nasralla et al. did not observe any statistical difference in occurrence of nonanastomotic biliary stricture in either groups [43, 45] (Table 5).

### 3.4. Post-Liver Transplant Survival Outcomes

During follow-up, 30 days' graft survival reported by three trials was between 90 and 100% in NMP group and 97.5 and 100% in SCS group [39, 43, 45]. Furthermore, 30 days' mortality ranged within 2.5–11% and within 0–2.5% in NMP and SCS group, respectively (Table 6).

### 3.5. Safety, Feasibility, and Logistics

None of the earlier four preliminary trials reported any issues related to device failure; however Nasralla et al. in a recent randomized trial (2018) did report single event of graft loss following device malfunction and two user related device errors [45]. Previously, Bral et al. reported loss of marginal DCD graft following unrecognized twist above the portal bifurcation led to initial perfusion failure [43]. Another technical complication, an airlock in the fluid sensing system encountered during transportation, was reported in the study by Ravikumar et al., necessitating transient stop for rectification [39] (Table 2). The study by Bral et al. observed a markedly prolonged ICU stay in the NMP group but attributed that largely to patient selection bias with greater preexisting comorbidities in the NMP group [43]. However, the authors could not discount the possibility that the NMP technology could have contributed in some manner, especially since they deliberately pushed the boundaries of perfusion times to the outer limits in some cases (up to 23 hours). Since all studies reported significantly lower reperfusion transaminases with NMP, one would anticipate that healthier livers with lower ICU stay; however, none of studies observed any difference in terms of ICU stay (Table 2).

## 4. Discussion

With the increasing incidence of the liver disease, the number of transplants required has been outpaced by the number of transplants performed. This disparity between liver transplant candidates and the availability of donor liver has led to an increase in mortality while waiting for transplantation. In order to meet the ever-increasing demand, transplant centres have started exploring the probability for utilization of marginal donor organs. However, the equilibrium has never been achieved owing to the compromised quality of such organs. Recent data from the Organ Procurement and Transplantation Network (OPTN) in the United States reveal that almost 22% of procured livers are discarded before transplantation, and likely many more are never offered as they are considered to have too high risk for cold storage. Currently, SCS technique is the mainstay of organ preservation. However this method works well for livers from healthy donors, achieving acceptable rates of EAD, PNF, and biliary complications [51, 52]. It does not hold true for marginal livers. Increasing evidence suggests that normothermic machine perfusion attempts to recreate the physiological environment by delivering oxygen, temperature, and nutrition. NMP has paved its way for the continuation of aerobic metabolism during the period of organ preservation by minimizing the effects of ischemia/reperfusion injury and PRS [53–56].

The efficacy of normothermic perfusion for liver preservation has not been fully described by clinical studies. In the present review, we have compiled all the evidence and shortcomings illustrated in the studies done so far. These trials were primarily done to assess the safety of NMP; however, data related to functioning and viability of organ were also included [39, 42, 43]. We note that there are very few clinical studies available to date, and thus far virtually all have been limited to safety and feasibility. Since the target population has generally been low-risk, more extensive studies in higher risk subjects will be needed to better define the full potential of this technology to enhance clinical outcomes and make unusable livers more usable for transplantation. Only future studies that focus on more marginal grafts will be able to define this potential more clearly.

### 4.1. NMP and Safety Issues

The primary aim of this review was to assess the safety of NMP by combining analyses of all available early phase clinical trials. The most frequently cited impediments to broader clinical significance of NMP include the unproven benefit and risk of this new technology, the potential substantial costs associated with complex portable machinery, complex procedures, and additional personnel required to tether and maintain liver stability during ex situ perfusion, risk of microbial contamination, and risk that good livers could be destroyed by exposure to instant warm anoxia if a device were to fail during transportation. Inadvertent incidents as graft loss following single event of unrecognized twist in portal vein or catheter occlusion in hepatic vein and bile duct or other user related errors could be a part learning curve associated with new technology and may get minimized with the greater cumulative team experience [43, 45, 49]. The real need for device portability remains undefined in clinical studies presently. Adequately powered randomized controlled head-to-head trials are needed to address many of these concerns. Portability allows for direct delivery of NMP at the donor centre with curtailment of cold ischemic exposure but substantially raises the complexity of moving large, heavy machinery by road or air transportation and requires additional technical staff to travel with the donor team. This would require engagement of additional or larger planes for transportation in donor centres that are more remote from the recipient transplant centre. The possibility of perfusing the procured liver once it arrives in the recipient centre (so-called “back-to-base”) could still potentially deliver many of the potential benefits of NMP but with far less cost and complexity. NMP technology does not eliminate reperfusion injury but rather brings that process ex situ such that, with appropriate predictive tools to assess function, the transplant surgeon may more accurately assess likelihood that a more marginal liver would work adequately in a particular recipient, before that recipient is exposed to that added risk. As more DCD livers are employed in clinical practice to address the acute liver donor shortage, balancing acute recipient need against potential escalating risk becomes critical. It remains to be seen whether NMP in its current form can protect against ischemic cholangiopathy (IC) in DCD donation. Since NMP does not prevent ischemic-reperfusion injury and biliary epithelial integrity cannot be readily assessed acutely based on currently available testing, we suspect that NMP will not eliminate risk of IC. Potentially, addition of antioxidants, caspase inhibitors, or other cellular protectants could ultimately protect human livers from such risk when added to NMP circuits, but this remains undefined presently.

The loss of clinically usable livers remains of potential concern if more centres are to face the learning curve of a more complex technology for liver preservation than the standard cold storage solution box, which is cheap, efficient, and of proven and known quantum over the past 40 years. It is possible that reporting bias may underreport technical errors in early studies.

A large body of large animal preclinical and now preliminary clinical data demonstrates that ex situ NMP based liver perfusion is generally safe, in most but not all cases lowers early transaminase levels in the recipient, and can accommodate a much prolonged storage phase than could safely be contemplated with SCS. However, adequately powered larger randomized studies are eagerly awaited to more fully define the risk-benefit balance, and until then the exact technique for this exciting new technology remains to be defined.

### 4.2. NMP and Prediction of the Viability of Donor's Liver

At present, the viability of any particular donor liver can only be assessed following the transplantation into a recipient, which could potentially prove disastrous when extreme marginal donor livers are transplanted, with elevated risk of PNF, EAD, or other detrimental effects of ischemic-reperfusion injuries, including PRS leading to acute renal injury [57–60].

NMP may help overcome such risks by allowing liver function assessment before implantation of the organ into a recipient. The benefits of NMP have been tested in numerous studies and suggest that the viability of donor organ can be predicted by a combination of hemodynamic, metabolic, and synthetic parameters derived during the ex situ perfusion phase, providing a functional assessment of the donor liver, which heretofore was not possible with SCS [34, 61]. The parameters assessed include bile production, stability of hepatic artery and portal flow and/or pressure, and other metabolic parameters as AST and ALT [39, 42, 43, 45]. The most potent predictors of adequate liver function posttransplant thus far are lactate clearance, pH stability, and need for repeated bicarbonate correction during NMP [33, 34, 62]. Mergental et al. suggested criteria including perfusate lactate < 2.5 mmol/l, bile production within 2 hours of initiation of NMP, pH > 7.3, hepatic artery flow > 150 ml/min, portal vein flow > 500 ml/min, and homogenous graft perfusion with soft parenchymal consistency fulfilled within 3 hours of initiation of NMP. Not all groups agree with this data, and one has to interpret any potential criterion within the context of which type of NMP or subnormothermic system they were developed within and within the constraints of which perfusates and additives were given, as it is likely that such criteria will not be universal across technologies (Table 7). Clearly, more detailed and extensive studies will be needed to cross validate such criteria.

### 4.3. NMP and Posttransplant Complications

Currently, up to one-third of the total pool of marginal livers are made up of DCD donor livers, even though such livers carry added risk [63–66]. While experimentally in porcine models the data is compelling that application of NMP can mitigate most of the added risk of the DCD donor, such data is currently lacking in clinical practice [64, 65]. Brockmann et al. found that NMP-perfused porcine DCD liver grafts have superior function and better survival compared with SCS [66]. Fondevila et al. found that porcine DCD livers exposed to a WIT up to 120 mins had 100% survival with NMP but 100% mortality with SCS [67]. The subsequent clinical trials published by Ravikumar, Selzner, Bral, and Nasralla et al. used 20%, 20%, 40%, and 24.8% DCD liver, respectively, and demonstrated similar outcomes to SCS controls in DCD grafts. Recent study by Nasralla et al. (2018) outlined better outcome with DCD livers preserved with NMP in comparison to SCS group; however further studies are required to strengthen this outcome owing to limitations of inadequately powered subgroup analysis [45].

Bral et al. did not show significant improvement in opening transaminase levels in the recipients, likely due to their increased proportion of DCD donors and prolonged cold ischemia time while a relatively higher proportion of replaced and accessory hepatic arteries were reconstructed on the back table, and their NMP duration was extended to outer limits often while their small surgical team rested overnight or were engaged in other hepatobiliary surgeries [43]. The primary safety outcomes were similar in the NMP group to SCS controls, and long term outcomes up to six months were comparable, suggesting potential benefit from NMP.

### 4.4. NMP and Posttransplant Primary Nonfunction (PNF)

Although SCS is considered as the gold standard method for liver preservation, the injurious impacts upon hepatocyte and biliary epithelial survival are well described [68, 69]. The combination of prolonged cold storage with a marginal liver graft may provide an insurmountable risk for a recipient [70–75]. PNF occurs in up to 5–8% of liver transplants and will result in recipient death if prompt retransplantation is not possible [76, 77]. Though PNF may be caused by technical failure resulting in inadequate blood flow through the graft [70], the association between excess donor risk factors and PNF suggests that it is likely multifactorial [71, 72]. None of the early phase NMP trials have thus far been associated with PNF.

### 4.5. NMP and Posttransplant Early Allograft Dysfunction (EAD)

Early allograft dysfunction (EAD) reflected by elevated recipient transaminase levels within the first postoperative week also poses increased risk for the recipient [78–80]. In the present systematic review EAD of clinical trials ranged from 10 to 55.5% with NMP. There were four incidents of EAD in Bral et al. study of these 2/3 that occurred in DCD grafts [39, 42–44]. All of these livers functioned well at the end and transaminase level returned to the baseline levels. In a recently completed randomized trial Nasralla et al. reported 93% less likelihood of developing EAD in DCD liver and improved graft functioning with NMP [45, 50].

### 4.6. NMP and Posttransplant Biliary Complication

Anastomotic biliary strictures or more diffuse IC are one of the most feared complications of DCD liver transplantation. The incidence of biliary strictures ranges between 4 and 15% following DBD liver transplantation while being within 30–40% after DCD [14, 74]. Injury to the peribiliary glands (PBG) following formation of microthrombi in peribiliary vascular plexus (PVP) at the time of ischemia-reperfusion or a circulatory phase of DCD has been implicated in biliary stricture formation [75, 81]. Later, Seal et al. reported that administration of a thrombolytic agent, tissue plasminogen factor (tPA), into hepatic artery dissolved thrombi in microcirculations and prevented any occurrence of thrombus and hence biliary strictures [82].

Op Den Dries et al. and Boehnert et al. reported significantly fewer bile duct related complications following NMP in the porcine model [25, 69]. Liu et al. demonstrated that biliary epithelium regeneration and differentiation of multipotent stem cells present in PBG into cholangiocytes after NMP in porcine model could prevent biliary strictures [83, 84].

However, subsequent clinical trials showed varied results as Bral et al. and Selzner et al. did not observe any late biliary complications despite the high number of DCD donors (40% and 20%, resp.), while Ravikumar et al. did report anastomotic strictures in 4 cases (20%) in the NMP group [39, 42, 43]. A recent randomized trial published by Nasralla et al. (2018) encountered similar rate of nonanastomotic strictures for DCD livers in NMP group 11.1% versus SCS 26.3% (*P* = 0.180). However, further research is warranted with NMP and DCD donation to more fully define risk and protection to the biliary epithelium.

### 4.7. NMP and Duration of Organ Preservation

Currently, the current median liver preservation time in the US is approximately 8 hours [85]. There are potential practical benefits if the preservation period can be more safely extended. Recently Vogel et al. showed successful liver transplantation after 48 hours of preservation on NMP device in their porcine model [78, 79]. The OrganOx Metra is currently licensed for clinical experimental study to preserve livers for up to 24 hours. In the study conducted by Ravikumar et al. one liver was perfused for 18.5 hours before being successfully transplanted [39]. Similarly, Bral et al. reported a DCD liver being successfully maintained for 22.5 hours on NMP before safe transplantation, although it should be noted in this case that the recipient sustained a prolonged period of cholestasis before eventual full recovery [43]. In a study by Nasralla et al. (2018) they observed significantly prolonged preservation in NMP group and reported significantly better early graft functioning with peak AST (NMP: 485 IU/L versus SCS: 974 IU/L; *P* < 0.0001) and EAD (NMP: 12.6% versus SCS: 29.9%; *P* = 0.002) [45, 50]. Hence, extended duration of preservation provides a more structured and orderly proposition for liver transplantation by promoting the judicious allocation of logistics as an assessing viability, operating room, staffing, and if required facilitating the preoperative optimization of the recipient. Additionally, there is potential that NMP could provide a window for introducing therapeutic interventions to further improve graft quality, and this requires more detailed study.

### 4.8. NMP in Liver Steatosis

Steatotic livers constitute a proportion of ECD grafts but have traditionally been discarded before transplantation due to their known increased risk for PNF [62, 80]. Spitzer et al. reported a 71% increased risk of 1-year graft loss with >30% macrosteatosis compared to controls with <15% steatosis [85]. Others have shown successful outcomes despite macrosteatosis > 30% provided that donor age is <40 years, CIT is < 11 hours, and donors were not DCD-derived [86–89]. Jamieson et al. (2011) reported substantial improvement in grade of steatosis in NMP perfusion of rat livers [30]. Others have demonstrated reduction in macrovascular steatosis with NMP alone or in association with defatting solutions [62, 90]. Nagrath et al. (2009) promoted the role of a “defatting cocktail” to esterify hepatic triglycerides and oxidation in steatotic rat livers with 65% reduction in hepatic triglyceride content [91]. However, Jamieson et al. found no evident reduction in NMP-perfused steatotic human livers, explained in part on the basis of inherent interspecies differences, fat solidification during obligate periods of cold storage, and total duration of perfusion (24 and 48 hours) [30, 91]. Further studies are required to clarify this issue and to optimize the role of defatting agents.

There are certain limitations to the present systematic review owing to a small number of studies available in the literature which might influence the interpretation of related outcomes. Despite these limitations, this systematic review has outlined the advantages of NMP over SCS in organ preservation and safety issues associated with its usage; however further randomized trials are much warranted to confirm these findings.

## 5. Conclusions

Over the past 40 years, SCS with refined cold preservation solutions has served the field well and has led to outstanding short and long term outcomes with clinical liver transplantation. As a result, liver transplantation remains a life-saving standard of care for all forms of acute, irreversible, and progressive chronic liver disease. The established success of this therapy and propagation of societal diseases such as hepatitis B, hepatitis C, and now widespread nonalcoholic steatohepatitis (NASH) had placed escalating pressure on transplant lists, driven supply-demand imbalance, and unacceptable rates of waiting list mortality. This situation has warranted a reevaluation of the methodology of storage and transportation of organs, as higher risk livers are used to attempt to match demand. Emerging evidence advocates that NMP may extend the safe utilization of the more marginal spectrum of liver donor grafts, but this remains to be proven in practice. This exciting technology has demonstrated safety and efficacy in preliminary clinical studies, and ongoing trials will continue to explore the full potential of NMP technologies, will determine the need for portability, and will more completely define the cost-benefit balance.

## Figures and Tables

**Table 1:** Criteria for the inclusion of studies.

Study design	Prospective study design with a well-defined study population

Study group	Liver transplant

Study size	Any

Length of follow-up	Any

Source	Peer-reviewed journals

Language	Any

Outcome measure	Patient safety, adverse events, graft function, Graft & patient survival and perfusion machine logistics.

**Table 2:** Pretransplant and perioperative characteristics of included studies.

Study	Sample Size (NMP vs Control)	Donor Age (years) (NMP vs Control)	MELD Score (NMP vs Control)	NMP Time vs Control SCS in minutes [median (range)]	DCD (NMP vs Control)
Ravikumar et al. [39] (May 2016)	20 vs 40	58.0 (21–85) vs 58.5 (21–82) (*P* = 0.93)	12.0 (7–27) vs 14.0 (6–25) (*P* = 0.55)	558 (210–1170) vs 534 (242–684) (*P* = 0.63)	4 vs 4
Selzner et al. [42] (October 2016)	10 vs 30	48.0 (17–75) vs 46.0 (22–68) (*P* = 0.56)	21.0 (8–40) vs 23.0 (7–37) (*P* = 0.85)	586 (221–731) vs 634 (523–783) (*P* = 0.11)	2 vs 6
Bral et al. [43] (Nov 2017)	10 vs 30	56.0 (14–71) vs 52.0 (20–77) (*P* = 0.91)	13.0 (9–32) vs 19.0 (7–34) (*P* = 0.37)	786 (304–1631) vs 235 (64–890) (*P* = 0.001)	4 vs 8
Liu et al. [44] (May 2017)	10 vs 40	NA (*P* > 0.05)	NA (*P* > 0.05)	(240–472) vs NA (*P* > 0.05)	2 vs 8
Nasralla et al. [45] (Apr 2018)	137 vs 133	56.0 (16–84) vs 56.0 (20–86) (*P* > 0.05)	13.0 (6–35) vs 14.0 (9–18) (*P* = NA)	714 (258–1527) vs 465 (223–967) (*P* < 0.000)	34 vs 21

MELD: model for end-stage liver disease; DCD: donation after circulatory death; NMP: normothermic machine perfusion; WIT: warm ischemia time; SCS: static cold storage; NA: not available.

**Table 3:** Characteristics during normothermic ex vivo liver perfusion of included studies.

Study	Perfusate	Peak AST (U/L)	Peak ALT (U/L)	Final lactate (mmol/L)	pH	Bile production (mL/hr)	Hepatic artery flow (mL/minutes)	Portal venous flow (mL/minutes)	Device failure	Major Technical Complication
Ravikumar et al. [39]	Gelofusine + 3-unit donor cross-matched PRBC	NA	NA	NA	NA (7.2–7.4)	NA	NA	NA	0	**0**

Selzner et al. [42]	Steen Solution + 3-unit PRBC	1647 (227–9200)	444 (152–1460)	1.46 (0.56–1.74)	7.26 (7.13–7.33)	7.6 (2.4–15.1)	300 (200–400)	1250 (1200–1300)	0	0

Bral et al. [43]	Gelofusine + 3-unit type “O” PRBC	NA	NA	NA	NA	6.2 (1.9–32.2)	NA	NA	0	1 (Single liver discarded due to portal vein twist)

Liu et al. [44]	Plasma + matched PRBC	NA	NA	NA	NA	NA (1–13)	NA	NA	0	0

Nasralla et al. [45]	Gelofusine + 3-unit donor cross-matched PRBC	NA	NA	NA	NA	NA	NA	NA	1 (Single liver discarded due to the pinch valve miscalibration causing hepatic artery hypoperfusion)	2

PRBC: packed red blood cells; AST: aspartate aminotransferase; ALT: alanine amino transferase; NA: not available.

**Table 4:** Clinical outcomes following normothermic ex vivo perfusion (NMP) of included studies.

Study	Peak AST, days 1–7, U/L, median (range) (NMP vs Control)	INR 1 week, median (range) (NMP vs Control)	Bilirubin 1 week, *μ*mol/L, median (range) (NMP vs Control)	ALP 1 week, U/L, median (range) (NMP vs Control)
Ravikumar et al. [39]	417 (84–4681) vs 902 (218–8786) (*P* = 0.03)	1.05 (0.88–1.40) vs 1.03 (0.90–2.22) (*P* = 0.92)	25 (8–211) vs 30 (9–221) (*P* = 0.20)	245 (81–568) vs 243 (76–743) (*P* = 0.79)

Selzner et al. [42]	619 (55–2858) vs 949 (233–3073) (*P* = 0.55)	1.1 (1–1.56) vs 1.1 (1–1.3) (*P* = 0.47)	25.6 (17.1–131.6) vs 47.53 (6.8–256.5) (*P* = 0.20)	202 (96–452) vs 147 (87–456) (*P* = 0.21)

Bral et al. [43]	1252 (383 to >2600) vs 839 (153 to >2600) (*P* = 0.52)	1.1 (1.1–1.6) vs 1.1 (0.9–1.5) (*P* = 0.44)	79 (17–344) vs 53 (8–340) (*P* = 0.35)	139 (40–626) vs 187 (58–524) (*P* = 0.62)

Liu et al. [44]	NA (*P* = 0.001)	NA	NA (*P* > 0.05)	NA

Nasralla et al. [45]	488.1 (408.9–582.8) vs 964.9 (794.5–1172.0) (*P* < 0.000)	1.24 (1.15–1.38) vs 1.24 (1.16–1.39) (*P* = 0.64)	38.5 (21.0–73.2) vs 49.1 (26.0–85.5) (*P* = 0.02)	NA

AST: aspartate aminotransferase; ALT: alanine amino transferase; ALP: alkaline phosphatase; NA: not available.

**Table 5:** Posttransplant outcomes of included studies.

Study	PNF *n* (%) (NMP vs Control)	EAD *n* (%) (NMP vs Control)	ICU stay days [median (range)] (NMP vs Control)	Hospital stay days [median (range)] (NMP vs Control)	Major Complications (Clavien-Dindo ≥ 3b) *n* (%) (NMP vs Control)	Biliary Complications (6 months) NMP *n* (%)
Ravikumar et al. [39]	0 (0) vs 0 (0) (*P* = 1.0)	3 (15) vs 9 (22.5) (*P* = 0.73)	3.0 (1–8) vs 3 (1–41) (*P* = 0.45)	12.0 (6–34) vs 14.0 (8–88) (*P* = 0.10)	NA	4 (20)

Selzner et al. [42]	0 (0) vs 0 (0) (*P* = 1.0)	NA	1.0 (0–8) vs 2 (0–23) (*P* = 0.54)	11.0 (8–17) vs 13.0 (7–89) (*P* = 0.23)	1 (10) vs 7 (23) (*P* = 0.5)	0 (0)

Bral et al. [43]	0 (0) vs 0 (0) (*P* = 1.0)	5 (55.5) vs 8 (29.6) (*P* = 0.23)	16.0 (2–65) vs 4 (1–29) (*P* = 0.004)	11.0 (8–17) vs 13.0 (7–89) (*P* = 0.23)	2 (22) vs 10 (37) (*P* = 0.6)	0 (0)

Liu et al. [44]	NA	1 (10%) vs 15 (36.8%) (*P* = 0.13)	NA	NA	NA	NA

Nasralla et al. [45]	1 (0.8) vs 0 (0) (*P* = NA)	12 (10.1%) vs 29 (29.9%) (*P* = 0.000)	4 (2–7) vs 4 (3–7) (*P* = 0.339)	15 (10–24) vs 15 (11–24) (*P* = 0.926)	21 (16.4) vs 36 (22) (*P* = NA)	13 (10.1)

PNF: primary nonfunction; EAD: early graft dysfunction; ICU: intensive care unit; NA: not available.

**Table 6:** Posttransplant survival outcomes of included studies.

Study	30 days of graft survival *n* (%)	3 months of graft survival *n* (%)	6 months of graft survival *n* (%)	Mortality *n* (%)
Ravikumar et al. [39]	20 (100) vs 39 (97.5) (*P* = 1.0)	NA	20 (100) vs 39 (97.5) (*P* = 1.0)	0 (0) vs 1 (2.5) (*P* = 1.0)

Selzner et al. [42]	NA	10 (100) vs 30 (100) (*P* = 1.0)	NA	0 (0) vs 0 (0) (*P* = NA)

Bral et al. [43]	9 (90) vs 30 (100) (*P* = 0.25)	9 (90) vs 30 (100) (*P* = 0.25)	8 (80) vs 30 (100) (*P* = 0.06)	2 (11) vs 0 (0) (*P* = 0.25)

Liu et al. [44]	NA	NA	NA	NA

Nasralla et al. [45]	116 (95.86) vs 99 (98.01) (*P* = 0.46)	NA	(95) vs (96) (*P* = 0.69)	NA

NA: not available.

**Table 7:** Mergental et al. viability criterion to define suitability of liver for transplantation.

Essential Parameters	Lactate < 2.5 mmol/L	OR	Bile Production
Any two of the following three criterions			
Perfusate pH > 7.3	Stable HA flow > 150 ml/min & PV flow > 500 ml/min		Homogenous graft perfusion with soft parenchymal consistency

## Abbreviations

ATP: Adenosine triphosphate
ALP: Alkaline phosphatase
ALT: Alanine aminotransferase
AST: Aspartate aminotransferase
INR: International normalized ratio
CIT: Cold ischemia time
DBD: Donors after brain death
DCD: Donor after circulatory death
ECD: Extended criteria donor
HA: Hepatic artery
IC: Ischemic cholangiopathy
IPF: Initial poor function
PNF: Primary nonfunction
PV: Portal vein
MAP: Mean arterial pressure
MELD: Model for End-Stage Disease
MSCs: Mesenchymal stem cells
NMP: Normothermic machine perfusion
PBG: Peribiliary gland
PVP: Peribiliary vascular plexus
PRS: Postreperfusion syndrome
SCS: Static cold storage
TPT: Total preservation time
Tregs: Regulatory T cells
WIT: Warm ischemia time.
